# Patient-Level Modeling of Ménière’s Disease vs. Vestibular Migraine: Performance of Speech Discrimination and Caloric-vHIT Dissociation

**DOI:** 10.3390/jcm15051908

**Published:** 2026-03-03

**Authors:** Nicolás Pérez-Fernández, Lorea Arbizu

**Affiliations:** 1 Department of Otorhinlarynglogy, Clínica Universidad de Navarra, Marquesado de Santa Marta 1, 28027 Madrid, Spain; 2 Department of Otorhinlarynglogy, Hospital Universtario de Navaarra, 31008 Pamplona, Spain

**Keywords:** Ménière’s disease, vestibular migraine, speech discrimination, caloric–vHIT dissociation, vHIT, calibration, decision-curve analysis, diagnostic modeling

## Abstract

**Background**: Differentiating Ménière’s disease (MD) from vestibular migraine (VM) remains difficult because current diagnostic frameworks are predominantly clinical and incorporate pure-tone thresholds, risking incorporation bias. We asked whether speech discrimination scores (SDS) alone can separate MD from VM at the patient level and whether adding a prespecified vestibular marker, the caloric–vHIT dissociation, pattern A (abnormal calorics with normal horizontal vHIT), improves performance. **Methods**: In a retrospective cohort (2015–2018) including definite MD (n = 60) and definite VM (n = 40) by Bárány/ICHD criteria, we trained patient-level logistic regression models with 5-fold out-of-fold validation and in-fold preprocessing. To avoid incorporation bias, PTA was excluded from all models. Predefined feature sets were as follows: (1) SDS-only (bilateral SDS), (2) CalHiT-A-only (Yes/No; canal paresis ≥22% with horizontal-canal vHIT gain ≥0.80 in either ear), and (3) SDS+CalHiT-A. Discrimination was assessed by ROC–AUC with bootstrap 95% CIs; calibration and decision-curve analysis (DCA) are reported. An exploratory model encoded SDS as “affected/healthy.” **Results**: The SDS-only model achieved AUC 0.866 (95% CI 0.787–0.937). CalHiT-A-only yielded AUC 0.674 (0.561–0.778). Adding CalHiT-A to SDS did not improve discrimination (SDS+CalHiT-A AUC 0.844 [0.760–0.913]). The exploratory “affected/healthy” SDS encoding underperformed (AUC 0.801 [0.706–0.882]). CalHiT-A was significantly more prevalent in MD than in VM (56.7% [34/60] vs. 17.5% [7/40]; Fisher’s exact *p* = 1.49 × 10^−4^). Calibration favored SDS-only, and DCA showed the highest net benefit for SDS-only across thresholds *p* = 0.05–0.40. **Conclusions**: Bilateral SDS alone provides robust, well-calibrated discrimination between MD and VM and outperforms CalHiT-A and the affected/healthy SDS encoding. In this cohort, vestibular test dissociation did not add diagnostic value beyond SDS at the patient level, supporting SDS-centered diagnostic workflows while reserving CalHiT-A for adjudication and phenotyping rather than primary classification.

## 1. Introduction

Accurately distinguishing Ménière’s disease (MD) from vestibular migraine (VM) remains, at times, one of the most demanding problems in contemporary neurotology [1]. Both disorders present with recurrent vertigo, yet they diverge in pathophysiology, prognosis, and treatment, so misclassification may lead to inappropriate management, delayed interventions, and worse outcomes [2]. Despite decades of research, reliable biomarkers that consistently separate MD from VM have proven elusive, although important advances have emerged in recent years [3]. Current diagnostic frameworks rely predominantly on clinical symptom profiles defined by the Bárány Society [4] and the International Headache Society [5], which are purely clinical classifications grounded in symptomatology and episode characteristics; given the overlap between syndromes, diagnostic ambiguity is frequent [6].

Current diagnostic frameworks for Ménière’s disease, including the 1995 Committee on Hearing and Equilibrium guidelines [7] and the 2020 AAO-HNSF Clinical Practice Guideline [8], emphasize pure-tone thresholds (0.5–3 kHz) and recommend concurrent assessment of speech recognition. However, the operational classification of hearing change and “usable hearing” remains primarily anchored in pure-tone averages, with speech discrimination used as a secondary modifier. Audiometry thus remains central to MD diagnosis together with symptomatology. In particular, pure-tone thresholds obtained during or shortly after attacks provide objective evidence of fluctuating sensorineural hearing loss, a defining feature of MD. This study extends that framework by formally integrating speech discrimination performance into the audiometric evaluation. By analyzing the relationship between PTA and SDS (through both correlation and derived indices such as the SDS/PTA ratio) the model captures functional hearing performance beyond sensitivity alone, potentially reflecting the degree of cochlear distortion characteristic of endolymphatic hydrops.

Recognition of the limitations of purely clinical definitions has also driven interest in objective physiological markers—beyond MRI—including advanced vestibular function tests [9] and emerging blood-based biomarkers [10]. By contrast, alternative MD classifications have integrated objective vestibular testing and hydrops MRI alongside clinical criteria, incorporating immediate nystagmus recording during attacks and gadolinium-enhanced MRI to visualize endolymphatic hydrops [11]. Early predictive models have been informative yet limited: a logistic approach suggested value in electrocochleography, head-shaking, ocular VEMPs, and horizontal-canal vHIT [12]; the Base-ML initiative tested multiple algorithms on registry data but achieved only 25.9–50.4% accuracies, underscoring the complexity of automated classification in this domain [13].

Among candidate physiologic markers, the dissociation between caloric and vHIT responses (abnormal calorics with preserved horizontal vHIT) has emerged as a reproducible MD signature [14,15], with large-scale studies supporting its specificity against VM and other vestibular disorders [16]. In this work, we refer to this dissociation as CalHiT-A (caloric–vHIT dissociation, pattern A). Notably, such caloric–vHIT dissociation has not been systematically integrated with speech measures and otolithic variables within a unified, leakage-controlled modeling framework.

Aligned with contemporary diagnostic frameworks [8,17], we designed a PTA-free analysis to minimize incorporation bias. Our primary focus was on bilateral speech discrimination scores (SDS, per ear), which capture functional hearing beyond pure-tone averages. We then asked whether vestibular physiology, prespecified as CalHiT-A, adds diagnostic information over SDS alone. Accordingly, our objectives were as follows:

- Primary objective: quantify the diagnostic performance of an SDS-only model (bilateral SDS per ear) for discriminating MD from VM using patient-level, out-of-fold cross-validation.
- Secondary objective: assess the incremental value of CalHiT-A over SDS by changes in ROC–AUC, and compare calibration and decision-curve net benefit; additionally, describe the prevalence of CalHiT-A by diagnostic group.
- Exploratory objective (pre-specified): compare ear-wise SDS to an “affected/healthy” SDS encoding and confirm that it does not outperform the bilateral specification.

All modeling used logistic regression with in-fold preprocessing; calibration and decision-curve analyses are summarized in the Supplementary Materials.

## 2. Material and Methods

### 2.1. Study Design and Participants

This was a cross-sectional, retrospective study including 60 patients with definite unilateral Ménière’s disease (MD) according to the 2015 Bárány Society criteria [4] and 40 patients with definite vestibular migraine (VM) according to the Bárány Society/ICHD consensus [5]. MD patients contributed both the affected and contralateral ears (120 ears total), while VM patients contributed both ears (80 ears total). The analysis used data from the index visit (2015–2018) only; subsequent visits were not used to compute predictors or outcomes.

Diagnostic stability was verified over 6–7 years using in-person clinic assessments whenever possible, with structured telephone interviews used only to supplement missing recent visits. No MD case progressed to bilateral involvement [18], and no VM case required diagnostic reclassification. This approach aligns with Bárány recommendations underscoring the role of longitudinal assessment to secure diagnostic accuracy in MD and VM. The study was approved by the institutional ethics committee and conducted in accordance with the Declaration of Helsinki. Given the retrospective design, informed consent was waived.

### 2.2. Patient-Reported Outcomes and Clinical Variables

All participants completed validated Spanish instruments at the index visit: the Dizziness Handicap Inventory (DHI) [19]; the Vertigo Symptom Scale (severity and anxiety subscales) [20]; in addition, patients completed the Cuestionario de Impacto Emocional del Vértigo (CIEV; Emotional Impact of Vertigo Questionnaire), originally developed and validated in Spanish [21]. We also recorded clinical features (auditory fluctuation, tinnitus, aural fullness, phonophobia, vascular triggers), disease duration (years), days since the last limiting vestibular event, and number of attacks in the preceding 12 months. A full otologic examination and a structured bedside vestibular evaluation (ocular motility, spontaneous nystagmus, post-head-shake nystagmus, positional testing) were performed. Ancillary evaluations (neurology, hydrops MRI) supported exclusion of alternative diagnoses and consolidation of MD or VM diagnoses as definite when needed.

These variables were used to characterize the cohort and for sensitivity/descriptive analyses; none were used as predictors in the primary models.

### 2.3. Audiologic Assessment

Pure-tone audiometry. Air- and bone-conduction thresholds were measured from 250 to 6000 Hz under standard clinical conditions. The conventional PTA was the mean at 0.5, 1, 2, and 3 kHz in line with AAO-HNS guidance [7]; a high-frequency PTA (2–6 kHz) was also computed [8].

Speech audiometry (SDS and SDS/PTA). Speech reception threshold (SRT) was the level (dB HL) yielding 50% correct for disyllabic words. Speech discrimination score (SDS) was obtained monaurally at a fixed presentation of 65 dB HL, using recorded, phonetically balanced disyllabic Spanish lists (Cárdenas-Marrero). Testing was performed in a sound-treated booth with calibrated transducers (insert or supra-aural as per clinic standards); 25 words/ear were presented once (no item repetition), and percent correct per ear was recorded. Masking with speech-spectrum noise was used when indicated by standard interaural-attenuation rules [22]. To quantify speech efficiency relative to tone loss, we computed SDS/PTA per ear as SDS (%)/PTA (dB HL, 0.5–3 kHz). This ratio operationalizes the classic observation that disproportionately reduced SDS relative to PTA suggests pathology beyond “amount of loss” and has long been used in diagnostic audiology [23]. Rationale for 65 dB HL: a fixed monaural level maximizes comparability across visits and aligns with conversational levels. If sensation level at 65 dB HL was low (e.g., SRT > 35 dB HL) or approached UCL, the presentation level was adjusted to SRT+35 dB HL or MCL, and the exact level was documented.

### 2.4. Vestibular Assessment

Calorics. Bithermal water irrigations (30 °C/44 °C) were performed, and the induced nystagmus was recorded using videonystagmography (VNG; ICS Natus, Middleton, WI, USA). Canal paresis (CP) was calculated according to Jongkees’ formula, with CP ≥ 22% considered abnormal.

Video head impulse test (vHIT). Horizontal and vertical semicircular canal VOR were measured by video-oculography (ICS Natus). Normal horizontal-canal gain was defined as ≥0.80; vertical ≥ 0.70.

VEMPs. cVEMP: sternocleidomastoid recordings using acoustic clicks and 100 Hz vibratory stimuli (Interacoustics, Middelfart, Denmark); P13/N23 latencies and amplitudes analyzed [24]. oVEMP: extraocular recordings to vibratory stimuli on the same platform; N10/P15 latencies and amplitudes analyzed [24].

Asymmetry ratio (AR): |Right − Left|/(Right + Left) × 100%.

Caloric–vHIT pattern taxonomy (CalHiT). We classified patient-level caloric–vHIT patterns using a simple 4-cell scheme (Table 1); the primary dissociation of interest is CalHiT-A. For the primary analyses, we encoded CalHiT-A (Yes/No) as the vestibular predictor (i.e., abnormal calorics with preserved horizontal vHIT in either ear). Other patterns were used for descriptive context and sensitivity checks.

### 2.5. Outcome, Data Structure, and Leakage Control

The outcome was diagnosis (MD = positive class; VM = negative class) at the patient level. To avoid leakage from side assignment, we did not use “affected ear” as a predictor; instead, ear-wise measurements entered the models as bilateral features (e.g., SDS-right, SDS-left). Pure-tone thresholds (PTA) were excluded from all models to minimize incorporation bias.

### 2.6. Feature Sets (Pre-Specified)

1. Primary (SDS-only): continuous SDS-right and SDS-left.
2. Secondary (CalHiT-A-only): Yes/No indicating CalHiT-A as defined above; if either component (calorics or vHIT) was missing, a Missing level was retained for descriptive reporting and sensitivity analyses (not treated as positive).
3. Tertiary (SDS + CalHiT-A): SDS-only with CalHiT-A added to assess incremental value.
4. Exploratory (ear-assignment check): affected/healthy SDS recoding where available, compared against the bilateral ear-wise specification.

Other available vestibular variables (e.g., CP% magnitude, per-ear vHIT gains) were summarized descriptively by diagnosis but not entered into the primary models.

Group comparisons. Demographic/clinical variables were compared between MD and VM: categorical variables by chi-squared test, continuous variables (non-normal distributions) by Mann–Whitney U. *p* < 0.05 was considered statistically significant.

Preprocessing, modeling, and validation. All preprocessing occurred within each training fold to prevent information leakage. Numeric features (SDS) used median imputation and z-standardization. Categorical features (CalHiT-A) used most-frequent imputation and one-hot encoding with unknown-level handling. The primary learner was logistic regression (liblinear, max_iter = 2000). Validation used 5-fold stratified out-of-fold (OOF) cross-validation at the patient level; OOF probabilities were aggregated for evaluation. Robustness checks repeated key analyses after (i) forcing CalHiT-A levels to Yes/No (excluding Missing from modeling), and (ii) confirming that affected/healthy SDS encoding did not outperform the bilateral SDS specification.

We prespecified a parsimonious logistic regression using two predictors anchored in clinical physiology (SDS and CalHiT-A) to minimize optimism and diagnostic circularity and to preserve interpretability. Given our events-per-variable constraints and the importance of probability calibration for clinical thresholds, we prioritized a well-calibrated linear model over higher-variance, flexible learners. All preprocessing (imputation/standardization) was performed in-fold to avoid leakage, and performance was estimated with out-of-fold predictions.

Performance metrics and statistical analysis. Discrimination was assessed by ROC-AUC with bootstrap 95% CIs (1000 resamples). Calibration (slope, intercept, Brier score) and decision-curve analysis (DCA) were computed on OOF predictions and are summarized in the Supplementary Materials. Threshold-based metrics (accuracy, sensitivity, specificity, PPV, NPV) are reported at *p* = 0.50 and Youden’s J in the Supplementary Materials. Counts of CalHiT-A (Yes/No) by diagnostic group and the Fisher exact *p*-value are provided in Results.

Software. All analyses were conducted in Python 3.10 with scikit-learn, following the pipeline above (in-fold preprocessing; OOF evaluation; bootstrap CIs) [25].

Statistical analyses involving multivariable machine learning models and assistance in language revision were supported by ChatGPT 4.0 (OpenAI, San Francisco, CA, USA). The authors main-tained full intellectual control over the content, and all outputs generated by the tool were critically reviewed, validated, and revised for accuracy and consistency with the study data.

## 3. Results

### 3.1. Demographic and Clinical Characteristics

A total of 100 patients were included: 60 with definite unilateral MD (120 ears) and 40 with definite VM (80 ears). Sex distribution differed significantly: VM showed a marked female predominance (87.5%) versus MD (55%, *p* < 0.001). Mean age did not differ (MD: 52.3 ± 12.1 years; VM: 48.7 ± 11.8 years; *p* = 0.15). Table 2 summarizes demographics, clinical features, and patient-reported outcomes; questionnaires indicated high disease activity at specialty referral.

### 3.2. Audiometric Findings

**Figure 1:**
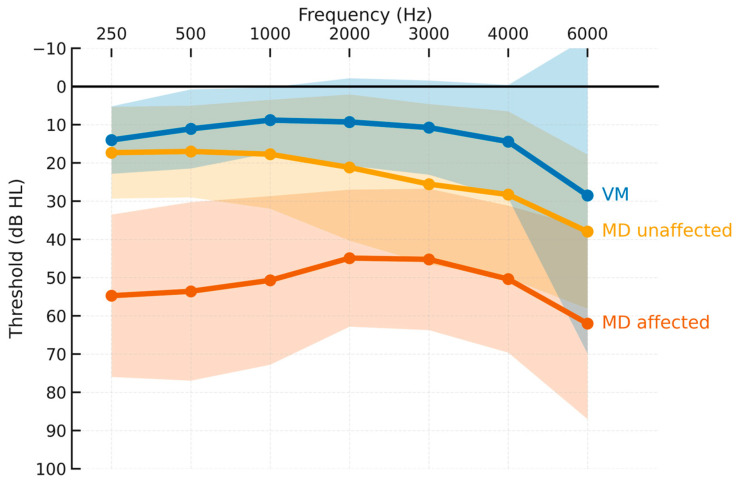
Group mean audiograms with SD ribbons. Mean air-conduction thresholds (dB HL) for MD-affected (vermillion), MD-unaffected (orange), and VM (blue) are plotted across 250, 500, 1000, 2000, 3000, 4000, and 6000 Hz. Shaded bands represent ±1 SD around the mean. The audiogram axis is oriented with −10 dB at the top and 100 dB at the bottom; a horizontal line marks 0 dB. Frequency tick marks are shown on the top axis, and group labels are placed inline at the end of each curve. Abbreviations: MD, Ménière’s disease; VM, vestibular migraine.

Across 250–6000 Hz (Figure 1), MD-affected ears showed the highest thresholds with the typical low- to mid-frequency predominance; VM ears remained near normal across most frequencies; MD-unaffected ears were intermediate (largely normal at low frequencies with a mild high-frequency slope). These gradients were consistent across frequencies and mirrored bar-plot contrasts at 250 Hz, PTA 0.5–3 kHz, and PTA 2–4 kHz.

**Figure 2:**
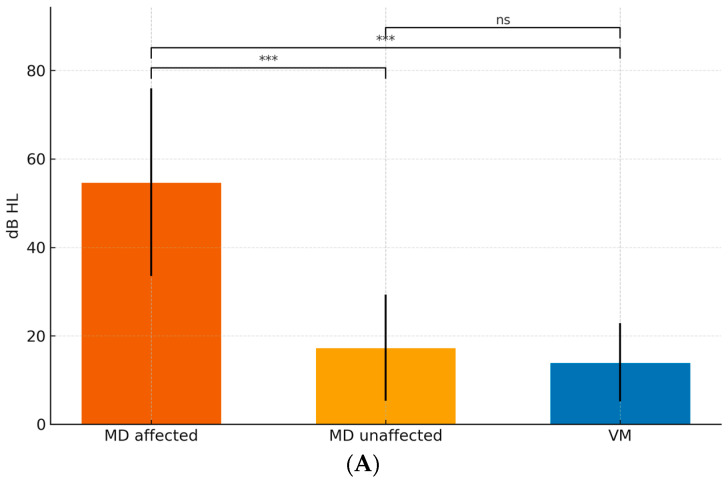

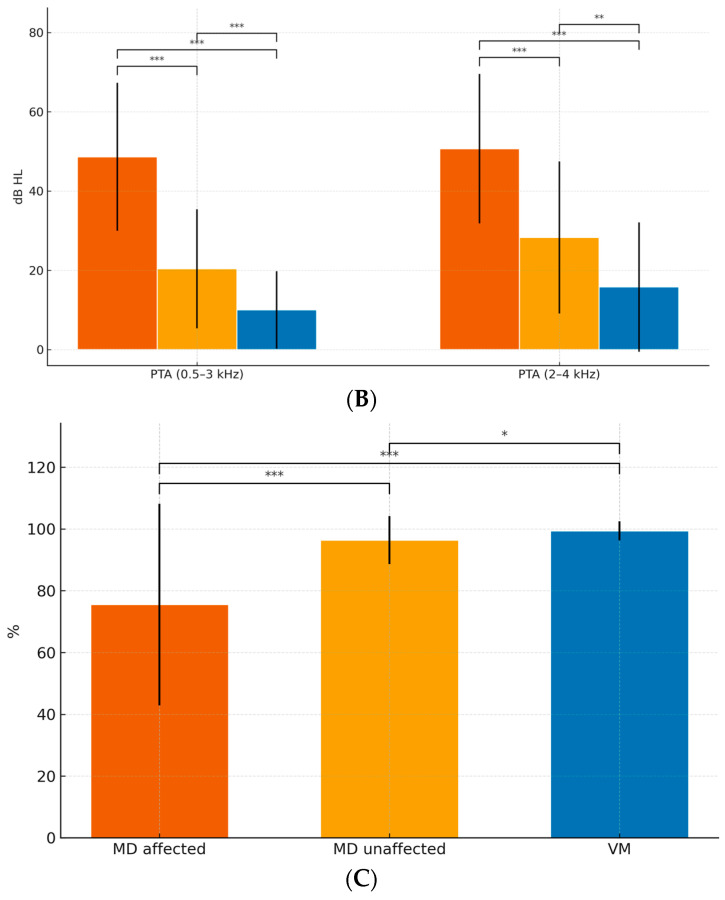
Group mean audiograms with SD ribbons. Bar charts display mean ± SD for (**A**) 250 Hz, (**B**) PTA 0.5–3 kHz and PTA 2–4 kHz, and (**C**) SDS (%). Significance markers (*, **, ***, “ns”) denote Bonferroni-adjusted two-sided permutation tests for all three pairwise comparisons (MD-affected vs. VM; MD-affected vs. MD-unaffected; MD-unaffected vs. VM). *X*-axis labels: MD-affected, MD-unaffected, VM; Vestibular migraine ears.

Considering all three groups (MD-affected, MD-unaffected, VM), Figure 2 shows that at 250 Hz thresholds were highest in MD-affected, intermediate in MD-unaffected, and lowest in VM; the same gradient held for PTA 0.5–3 kHz and PTA 2–4 kHz. Speech discrimination (SDS, %; 65 dB HL) was lowest in MD-affected, intermediate-to-high in MD-unaffected, and near ceiling in VM. Pairwise, permutation-based tests with Bonferroni correction (Table 3) indicated the following:

- MD-affected vs. VM: consistently large differences at 250 Hz, PTA 0.5–3 kHz, PTA 2–4 kHz, and SDS (all p_adj < 0.001).
- MD-affected vs. MD-unaffected: higher thresholds across tones and lower SDS in MD-affected (all p_adj < 0.001).
- MD-unaffected vs. VM: higher thresholds and lower SDS in MD-unaffected; differences remained significant after multiplicity control.

These findings support a graded audiometric profile (VM ≈ near-normal, MD-unaffected intermediate, MD-affected worst) mirrored by SDS, jointly providing robust separation across groups.

We found that although men had lower SDS scores than women when considering both sides, the main factor was diagnosis—MD versus VM—rather than sex, which is skewed toward females in VM. Without diagnosis (age-adjusted). Worst ear SDS: females outperform males by 13.4% (β = +13.41, *p* = 0.016); age effect −0.62%/year (*p* < 0.001). Bilateral mean SDS: females +7.1% (β = +7.08, *p* = 0.016); age −0.38%/year (*p* < 0.001). Best ear SDS: no significant sex effect (β = +0.76, *p* = 0.53).

With diagnosis (age-adjusted; MD vs. VM included). Interaction sex × diagnosis: not significant (SDS_worst *p* = 0.65; SDS_mean *p* = 0.77). Main sex effect (male vs. female): attenuates and becomes non-significant as SDS_worst is −6% (95% CI −17.5 to 5.5; *p* = 0.30); SDS_mean is −3.5% (95% CI −9.2 to 2.2; *p* = 0.23). Diagnosis effect (VM vs. MD): VM higher SDS: SDS_worst +18.0 (95% CI +6.3 to +29.6; *p* = 0.0029); SDS_mean +9.1 (95% CI +2.9 to +15.2; *p* = 0.0044). Age: remains negative (SDS_worst ≈ −0.48/year, *p* = 0.004; SDS_mean ≈ −0.30/year, *p* = 0.006).

**Figure 3:**
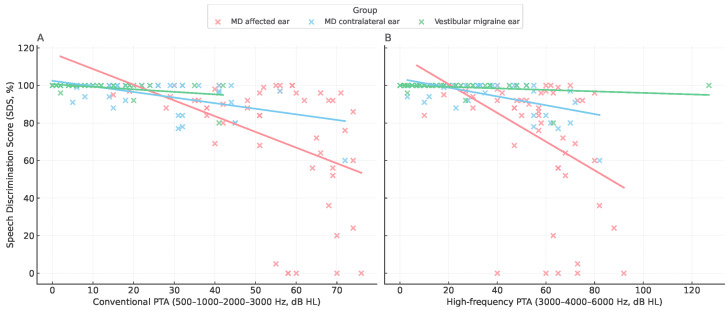
Scatterplots of SDS versus PTA. (**A**) Speech discrimination score (SDS) plotted against conventional PTA (500–1000–2000–3000 Hz). (**B**) SDS plotted against high-frequency PTA (3000–4000–6000 Hz). Red dots represent MD-affected ears, blue dots represent MD contralateral ears, and green dots represent VM ears. Linear regression lines are shown for each group.

**Figure 4:**
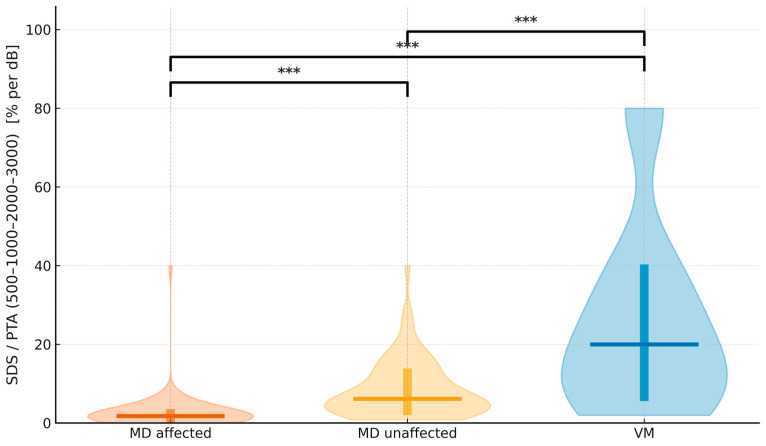
Violin plots show the distribution of SDS/PTA (500–1000–2000–3000) expressed in % per dB for MD-affected (vermillion), MD-unaffected (orange), and VM (blue). A horizontal line marks the median, and a thick vertical bar indicates the IQR (25th–75th percentiles) for each group. Brackets with asterisks denote Bonferroni-adjusted, two-sided permutation tests for the three pairwise comparisons (*** *p* < 0.001). The plotting range is limited to the 1st–99th percentiles for clarity; no observations were excluded from the statistical analysis.

**Figure 5:**
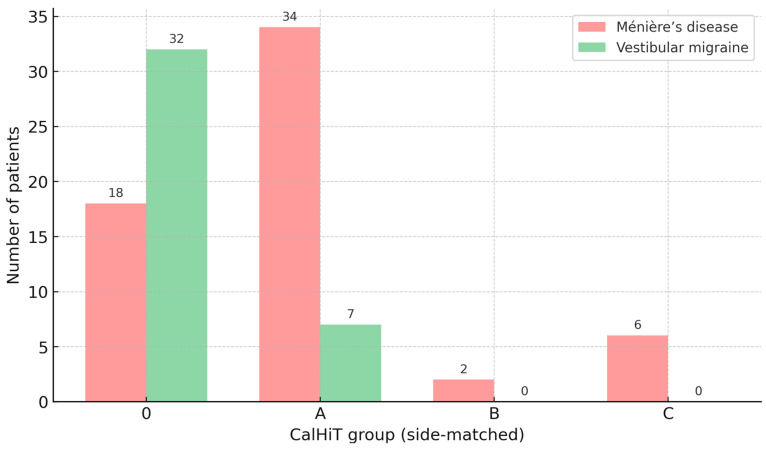
Diagnosis distribution by CalHiT group (side-matched), counts. Bars show the number of patients per CalHiT group for Ménière’s disease (soft red) and vestibular migraine (soft green). Side-matched rule: the caloric lesser-function side is compared with the vHIT of the same side. CalHiT-0 = normal calorics and normal same-side horizontal vHIT; CalHiT-A = canal paresis ≥ 22% with same-side vHIT gain ≥ 0.80; CalHiT-B = normal calorics with same-side vHIT gain < 0.80; CalHiT-C = study-specific atypical category.

The relationship between speech discrimination (SDS) and tonal thresholds (PTA, 0.5–3 kHz) revealed a consistent inverse trend across diagnostic groups (Figure 3). Higher PTA values were associated with lower SDS, but the slope of this relationship differed markedly as it was steeper in MD-affected ears and shallower in vestibular migraine, indicating group-specific patterns of auditory distortion (Figure 4, Table 4). To express this relationship in functional terms, we derived the SDS/PTA ratio, which estimates speech efficiency. As illustrated in Figure 5, this ratio was highest in vestibular migraine, intermediate in MD-unaffected ears, and lowest in MD-affected ears. All three pairwise contrasts remained statistically significant after Bonferroni-adjusted, two-sided permutation testing. For visualization only, values were truncated to the 1st–99th percentiles, whereas all statistics were performed on the complete dataset.

### 3.3. Vestibular Findings

- Canal function (calorics and vHIT)

In MD (n = 60), the predominant class was CalHiT-A (56.7%, 34/60), followed by CalHiT-0 (30.0%, 18/60), CalHiT-C (10.0%, 6/60), and CalHiT-B (3.3%, 2/60). In vestibular migraine (n = 40), CalHiT-0 dominated (80.0%, 32/40), with CalHiT-A at 17.5% (7/40) and CalHiT-B at 2.5% (1/40); no CalHiT-C cases were observed (see Figure 5).

Canal paresis (CP%) differed markedly by class: CalHiT-0 mean 11.36 (SD 5.52; median 10.5; n = 50); CalHiT-A 46.46 (19.92; 45.0; n = 41); CalHiT-B 13.33 (9.81; 19.0; n = 3); CalHiT-C 54.67 (26.50; 50.0; n = 6). Canal paresis (CP%) by diagnosis. MD: mean 35.05 (SD 21.90; median 31.5; n = 60). VM: mean 18.45 (SD 22.00; median 10.0; n = 40). Statistical contrasts (CP%). MD vs. VM (Mann–Whitney U): U = 1896.5, *p* = 9.58 × 10^−7^ ⇒ CP% is significantly higher in MD than VM. Across CalHiT classes (Kruskal–Wallis): H = 74.36, *p* = 4.98 × 10^−16^ ⇒ CP% differs significantly across rule categories.

As expected, VM concentrates in CalHiT-0 (normal calorics and normal same-side vHIT), whereas MD shows a predominance of CalHiT-A (abnormal calorics with preserved same-side vHIT), with low CP% in CalHiT-0/B and high CP% in CalHiT-A/C.

- Otolithic function (VEMPs)

We compared inter-aural amplitude ratios (IAAR, %) between MD and VM across click cVEMP, 100 Hz vibratory cVEMP, and vibratory oVEMP. IAAR distributions were centered near 0% with broad dispersion in both groups; values beyond ±40% occurred in each cohort. Two-sided permutation tests (10,000 permutations), Bonferroni-adjusted across modalities, showed no significant MD–VM differences (ns for all). Thus, IAAR did not discriminate MD from VM in this sample (Supplementary Table S1, Figure S1).

### 3.4. Multimodal Diagnostic Models

**Figure 6:**
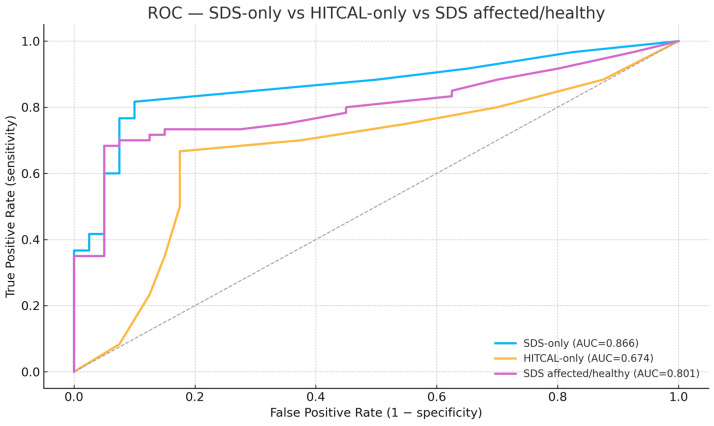
ROC curves comparing models based on SDS (SDS-only), SDS encoded as affected vs. healthy ear, and the caloric–vHIT dissociation (HITCAL). The SDS-only model shows the best discrimination, followed by the affected/healthy SDS encoding, while HITCAL alone performs more modestly. Curves are out-of-fold predictions from 5-fold stratified logistic regression with in-fold imputation and standardization; HITCAL was one-hot encoded. PTA variables were excluded. (AUC reflects discrimination: 0.5 = chance, 1.0 = perfect.).

Overall diagnostic performance (primary analysis; logistic regression, 5-fold OOF CV) —Figure 6.

- SDS-only (bilateral SDS per ear): ROC–AUC 0.866 (95% CI 0.787–0.937; n = 100).
- CalHiT-A-only (Yes/No): ROC–AUC 0.674 (0.561–0.778).
- SDS + CalHiT-A: ROC–AUC 0.844 (0.760–0.913)—no improvement over SDS alone (overlapping CIs; no practical gain).

### 3.5. Exploratory SDS Encoding

Re-expressing SDS as “affected/healthy” (where available) underperformed the bilateral ear-wise specification (AUC 0.801 [0.706–0.882]). The combined SDS (affected/healthy) + CalHiT-A model remained inferior (AUC 0.816 [0.726–0.893]).

### 3.6. Prevalence Context

As above, CalHiT-A was present in 70.7% of MD (41/58) vs. 16.7% of VM (9/54), *p* = 1.56 × 10^−6^, indicating strong physiologic separation at the group level despite the lack of incremental AUC when added to SDS in patient-level models.

Calibration and decision analysis. Calibration and decision-curve analyses are summarized in the Supplementary Materials; neither altered the primary conclusion that SDS alone provides the highest and most parsimonious discrimination, with no consistent benefit from adding CalHiT-A.

## 4. Discussion

Distinguishing Ménière’s disease (MD) from vestibular migraine (VM) remains challenging because prevailing diagnostic frameworks are largely clinical and place heavy weight on pure-tone thresholds thus creating a risk of incorporation bias when PTA is also used as a predictor. Within this context, our work shows that bilateral speech discrimination scores (SDS, per ear) provide strong, well-calibrated discrimination between MD and VM without circularity, and that the simple SDS/PTA ratio adds a functional perspective beyond tone thresholds. In parallel, the caloric–vHIT dissociation, pattern A (CalHiT-A: abnormal calorics with preserved same-side horizontal vHIT, side-matched) captures a vestibular phenotype highly characteristic of MD at the group level and clinically valuable for adjudication and phenotyping even if, in our dataset, it did not improve AUC once SDS was in the model. We therefore propose a dual-axis diagnostic paradigm: Audiologic (SDS/SDS-PTA) for primary classification, and Vestibular (CalHiT-A) for adjudication and phenotype definition.

Previous research has emphasized the divergence between PTA and SDS in Ménière’s disease [26]. Current guidelines prioritize documenting fluctuation through serial PTA measurements (ideally obtained during or shortly after attacks) and, in practice, rely on tonal thresholds for longitudinal monitoring. While indispensable for defining auditory change, this emphasis entails two limitations: (i) it risks circularity, as PTA values contribute both to diagnostic criteria and prognostic modeling; and (ii) it underrepresents the functional dimension that matters most to patients—speech understanding. In the present study, we intentionally excluded PTA from predictive modeling to minimize incorporation bias and demonstrated that bilateral SDS alone achieved high discrimination and robust calibration relative to vestibular markers. Re-expressing SDS as a simple “affected/healthy” difference did not outperform the bilateral, ear-wise representation.

Speech audiometry has long been acknowledged within the diagnostic framework of Ménière’s disease. The 1972 Committee on Hearing and Equilibrium already recognized its contribution to evaluating functional hearing [27]. The 1985 revision went further, explicitly requiring the use of word recognition scores (WRS) alongside pure-tone audiometry to define clinically significant hearing changes, setting a ≥15% difference in WRS as the criterion for both diagnosis and therapeutic follow-up [28]. Subsequent iterations [8,17] maintained the inclusion of speech testing but gradually relegated it to a secondary role, emphasizing tonal thresholds as the principal benchmark for progression. Our results revive and extend that early vision: by demonstrating that bilateral SDS alone discriminates effectively between MD and VM and that speech efficiency (SDS/PTA) systematically distinguishes affected from unaffected ears, this study provides empirical support for reinstating speech audiometry as a core diagnostic element. Rather than a discretionary adjunct, it should be regarded as integral to the audiologic phenotype of Ménière’s disease.

In our cohort, men appeared worse on SDS when diagnosis was ignored; however, once MD vs. VM was included, diagnosis dominated and the residual sex difference was small and not statistically significant. Within MD there was a non-significant trend toward lower SDS in men (7%; confidence intervals overlapped), while within VM sex differences were negligible, and the sex × diagnosis interaction was not significant. Taken together, these findings suggest that apparent sex differences in SDS largely reflect disease mix and age rather than sex per se, reinforcing the need to report SDS with diagnostic stratification and appropriate adjustment, and to avoid over-interpreting crude sex contrasts.

The SDS/PTA (0.5–3 kHz) ratio in this study operates as a speech-efficiency index: for a given amount of tonal loss, how effectively does the patient recognize words? Importantly, SDS and PTA are associated but PTA does not deterministically set SDS as intelligibility depends on more than audibility (e.g., distortion, recruitment, temporal and suprathreshold factors). We therefore use SDS/PTA as a descriptive index (not a model predictor) to express “speech per unit of tone loss”. In our cohort, SDS/PTA showed a graded pattern (VM highest, MD-unaffected intermediate, MD-affected lowest), indicating that the decline in intelligibility in MD exceeds what would be expected from thresholds alone. The metric is simple and readily standardized (fixed presentation level; recorded, equivalent lists; predefined 0.5–3 kHz band). This builds on a well-established audiologic principle: the SDS–PTA relationship has long been used to flag retrocochlear involvement when speech discrimination is disproportionately poor relative to mild pure-tone loss, helping distinguish cochlear from retrocochlear pathology [29] and we previously have shown how it evolves in follow-up MD [22]. We advocate routine reporting of SDS/PTA alongside SDS and PTA to better capture the functional footprint of MD and to enable clean longitudinal tracking of functional fluctuation.

Between attacks, vestibular migraine generally shows near-normal pure-tone thresholds with ceiling-level SDS, whereas Ménière’s disease shows depressed (often asymmetric) SDS. An SDS-centered workflow, qualified by SDS/PTA, therefore resolves much of the diagnostic ambiguity without invoking predictors embedded in the case definition. That a purely tonal model performs well is unsurprising given current criteria; the clinically meaningful advance is that SDS performs as well or better without circularity and with superior calibration.

CalHiT-A (abnormal calorics with preserved same-side horizontal vHIT; side-matched rule) reflects the frequency–time dissociation between tests that do also reflect different grounds: caloric is a test of nystagmus while vHIT is a test of vestibulo-ocular reflex. It aligns also with known hydropic mechanics [30,31]. In our sample, CalHiT-A was far more prevalent in MD than in VM (56.7% [34/60] vs. 17.5% [7/40]; Fisher’s exact *p* = 1.49 × 10^−4^), underscoring group-level specificity. Although adding CalHiT-A to SDS did not increase AUC in patient-level models, CalHiT-A does add clinical value: it (i) adjudicates borderline cases when SDS/SDS-PTA are equivocal, (ii) defines phenotypes (e.g., MD–CalHiT-A) with potential prognostic and therapeutic implications, and (iii) documents vestibular physiology in a disease where vertigo attacks drive much of the disability while VM is defined by burdensome vestibular symptoms. For these reasons, CalHiT-A should be explicitly included in diagnostic pathways, as a minimum recommended vestibular criterion (at least once during work-up) and as a phenotypic descriptor [16,32,33,34].

Caloric nystagmus results from unnatural stimulation akin to low-frequency rotation (~0.003 Hz) and is affected by velocity storage and central modulation. In contrast, vHIT measures the vestibulo-ocular reflex at high frequencies (~5–7 Hz). Thus, reduced caloric responses with normal vHIT gains are possible, especially between attacks in VM patients: 33.9% of patients with abnormal calorics had normal vHIT, while 11.9% with abnormal vHIT had normal calorics [33]. Previous data were similar (28% vs. 6% [35]. A recent study found that having normal vHIT but pathological calorics helps distinguish MD from VM (specificity ~83.5%, sensitivity ~58.9%), though this pattern can occur in both conditions [32].

In our cohort, inter-aural amplitude ratios (IAAR) for click cVEMP, 100 Hz vibratory cVEMP, and vibratory oVEMP were centered near 0% with broad dispersion in both MD and VM. Given this lack of discriminatory signal at the univariate level, we did not include VEMPs in the predictive models. This preserves parsimony and calibration, avoids penalizing patients with missing otolithic data, and prevents diluting the clear audiologic and canal-level vestibular effects (SDS/SDS-PTA and CalHiT-A). Methodologically, VEMP amplitudes are susceptible to high physiological and technical variance (age effects, sternocleidomastoid activation, montage, conductive factors, and time since the last vertigo spell or activity of the disease), which can obscure modest between-diagnosis differences. Clinically, our results suggest that VEMPs add limited value for the binary MD-VM separation, although they remain useful for otolithic phenotyping, longitudinal tracking, and specific differential diagnoses outside the present scope. Future work could revisit VEMPs using EMG-normalized metrics, frequency-tuning paradigms, or interaction-aware models (e.g., coupling VEMP features to depressed SDS or low SDS/PTA), but on current evidence they should not be part of the primary MD–VM classifier.

In keeping with AAO-HNS/Bárány frameworks that already stress documenting fluctuation with PTA, we propose three concrete additions for future updates: (1) make bilateral SDS mandatory for diagnosis and follow-up of MD, alongside PTA/SRT, using a standardized protocol (recorded, equivalent lists; stable presentation level); (2) report SDS/PTA (0.5–3 kHz) routinely as an efficiency index to enable inter-center comparability and longitudinal interpretation anchored to function; (3) adopt CalHiT-A (side-matched caloric/vHIT dissociation) as a recommended vestibular criterion and as a phenotypic label (e.g., MD–CalHiT-A±), obtained with calorics + vHIT at least once and repeated when clinical decisions hinge on vestibular status—i.e., a shift from “PTA-centric” to “dual-axis.”

It is important to note that our analyses were conducted under a specific, historically grounded audiometric methodology [36]. For speech discrimination testing we recommend two practical implementation options: (1) a two-step fixed-level rule presenting SDS monaurally at 65 dB HL with recorded, equivalent lists via inserts/supra-aurals; if the sensation level is low (e.g., SRT > 35 dB HL) or approaches UCL, switch to SRT+35 dB HL or MCL; or (2) a single-step variable-level rule presenting SDS at SRT+35 dB HL (or MCL) from the outset. We used the former rule in this study because it maximizes comparability across visits and aligns with conversational levels; whichever option is chosen, we recommend documenting the exact presentation level, transducer, list ID, and masking.

Strengths include an analysis free of incorporation bias (PTA excluded from modeling), patient-level, out-of-fold validation, and pre-specified comparisons across SDS, CalHiT-A, and SDS+CalHiT-A with calibration and decision-curve evaluation as currently ongoing [37]. Limitations include the retrospective, single-center design; use of Spanish recorded lists at a fixed level; absence of hydrops MRI as a predictor in the primary model; and interictal testing (peri-attack speech testing could increase sensitivity). Regarding the first issue we used a constrained feature set by design; more flexible learners may prove advantageous in larger, multi-center datasets with additional predictors. External validation and pre-registered algorithm comparisons are planned.

## 5. Conclusions

Bilateral speech audiometry (with SDS as the anchor and SDS/PTA as a complementary index) should be part of the diagnostic and follow-up core for Ménière’s disease because it offers high, non-circular discrimination and maps to functional outcomes. CalHiT-A is necessary to capture vestibular physiology, adjudicate ambiguous cases, and define clinically useful phenotypes. A dual-axis strategy (SDS/SDS-PTA for primary classification and CalHiT-A for adjudication/phenotyping) provides a pragmatic, patient-centered path ready for clinical adoption and worthy of inclusion in future guidelines.

Notwithstanding these strengths, the present evidence comes from a single-center cohort and requires external validation across multiple sites (ideally with harmonized protocols) to confirm generalizability and clinical utility.

## Figures and Tables

**Table 1:** CalHiT pattern taxonomy (patient-level).

Code	Plain Name	Definition (Either Ear Qualifies)
CalHiT-0	Concordant normal	Canal paresis (CP) < 22% and horizontal vHIT gain ≥ 0.80
CalHiT-A	Dissociation A (calorics abnormal, vHIT normal)	CP ≥ 22% and horizontal vHIT gain ≥ 0.80
CalHiT-B	Dissociation B (calorics normal, vHIT abnormal)	CP < 22% and horizontal vHIT gain < 0.80
CalHiT-C	Concordant abnormal	CP ≥ 22% and horizontal vHIT gain < 0.80

Notes: (1) CP computed by Jongkees’ formula; threshold for abnormality set at ≥22%. (2) Horizontal-canal vHIT abnormality threshold set at gain <0.80; vertical canals were not used in CalHiT coding.

**Table 2:** Demographic and clinical characteristics of the study population. Data are presented as n/total for categorical variables and mean ± standard deviation for continuous variables. *p*-values were obtained using chi-squared test (categorical) or Mann–Whitney U test (continuous).

Variable	MD (n = 60)	VM (n = 40)	*p*-Value
Hearing fluctuation	60/60	0/40	nan
Tinnitus	**60/60**	3/40	0.000
Pressure	**57/60**	7/40	0.000
Noise intolerance	**51/60**	11/40	0.000
Vascular risk factors (>2)	17/60	26/40	0.001
Tumarkin crises	6/60	1/40	0.298
Headache	11/60	**40/40**	0.000
Photophobia	2/60	**27/40**	0.000
Phonophobia	3/60	**28/40**	0.000
Spontaneous nystagmus	6/60	6/40	0.660
Post-head-shake nystagmus	10/60	14/40	0.062
DHI	42.8 ± 18.4	40.9 ± 27.3	0.556
VSSS	14.2 ± 10.4	12.4 ± 7.4	0.683
VSSA	11.1 ± 7.4	14.2 ± 8.7	0.074
CIEV	13.0 ± 6.2	15.2 ± 9.0	0.357
Disease duration (years)	**5.2 ± 6.0**	3.4 ± 3.8	0.043
Days since last vertigo spell	54.7 ± 83.8	64.3 ± 85.0	0.288
Number of crises in last year	7.2 ± 12.1	8.8 ± 12.3	0.941

MD, Ménière’s disease; VM, vestibular migraine; DHI, Dizziness Handicap Inventory; VSSS, Vertigo Symptom Scale severity, VSSA, Vertigo Symptom Scale anxiety; CIEV, Emotional Impact of Vertigo Questionnaire.

**Table 3:** Audiometry and speech discrimination by group. Means ± SD per group.

Metric	MD-Affected (n)	MD-Unaffected (n)	VM (n)	MD-Affected (Mean ± SD)	MD-Unaffected (Mean ± SD)	VM (Mean ± SD)	*p*-Value MD-Affected vs. MD-Unaffected	*p*-Value MD-Affected vs. VM	*p*-Value MD-Unaffected vs. VM
250 Hz (dB HL)	60	60	72	54.8 ± 21.2	17.3 ± 12.0	14.0 ± 8.8	<0.001 ***	<0.001 ***	0.213 ns
PTA 0.5–3 kHz (dB HL)	60	60	72	48.6 ± 18.7	20.4 ± 15.0	10.0 ± 9.8	<0.001 ***	<0.001 ***	<0.001 ***
PTA 2–4 kHz (dB HL)	60	60	72	50.7 ± 18.9	28.3 ± 19.2	15.8 ± 16.3	<0.001 ***	<0.001 ***	<0.001 ***
SDS (%)	60	60	50	75.5 ± 32.7	96.4 ± 7.8	99.4 ± 3.1	<0.001 ***	<0.001 ***	0.026 *

Notes: Means ± SD per group. Two-sided permutation tests (10,000 permutations), Bonferroni-adjusted across the three pairwise comparisons per metric. Significance: *** <0.001; * <0.05; ns = not significant.

**Table 4:** Linear regression equations for SDS versus PTA.

Panel	Group	Slope	Intercept	R^2^
Conventional PTA	MD-affected ear	−0.84	117.1	0.25
Conventional PTA	MD contralateral ear	−0.3	102.5	0.32
Conventional PTA	Vestibular migraine ear	−0.13	100.6	0.2
High-Frequency PTA	MD-affected ear	−0.76	116.0	0.24
High-Frequency PTA	MD contralateral ear	−0.24	103.6	0.36
High-Frequency PTA	Vestibular migraine ear	−0.04	100.0	0.08

Abbreviations: SDS, speech discrimination score; PTA, pure-tone average. Linear regression equations are expressed as y = slope × PTA + intercept. Coefficients of determination (R^2^) indicate the proportion of variance in SDS explained by PTA.

## Data Availability

The raw data supporting the conclusions of this article will be made available by the authors on request.

## Supplementary Materials

The following supporting information can be downloaded at: https://www.mdpi.com/article/10.3390/jcm15051908/s1, Figure S1: Side-by-side violin plots display IAAR (%) for AcVEMP, VcVEMP, and VoVEMP in MD (vermillion) and VM (blue). Each violin shows the median (horizontal line) and IQR (thick vertical bar). The y-axis is fixed to −100 to +100%, with dashed red lines at ±40% as pathological thresholds. Brackets with asterisks summarize MD–VM contrasts using two-sided permutation tests (10,000 permutations) with Bonferroni correction across modalities (* *p* < 0.05; ** *p* < 0.01; *** *p* < 0.001; ns = not significant). No legend is shown to maximize plotting area; Figure S2: Calibration (reliability)—SDS-only, HITCAL-only, and SDS affected/healthy; Figure S3: Decision-curve analysis—SDS-only, HITCAL-only, and SDS affected/healthy; Table S1: IAAR (%) in MD vs. VM across VEMP modalities; Table S2: Calibration metrics (out-of-fold); Table S3: Decision-curve net benefit by threshold (*p* = 0.05–0.40); Table S4: HITCAL counts by diagnosis and Fisher’s exact test.
