# Supplementary material for: Patient-Level Modeling of Ménière’s Disease vs. Vestibular Migraine: Performance of Speech Discrimination and Caloric-vHIT Dissociation
Source: J Clin Med. 2026 Mar 3;15(5):1908. doi: 10.3390/jcm15051908 (PMC12986367; doi:10.3390/jcm15051908)
Supplement: Supplementary file 1 [file jcm-15-01908-s001.zip › jcm-3956183-supplementary.pdf]

Supplementary material

1. <u>Otolithic function in the study</u>

Figure S1. Side-by-side violin plots display IAAR (%) for AcVEMP, VcVEMP, and VoVEMP in MD (vermillion) and VM (blue). Each violin shows the median (horizontal line) and IQR (thick vertical bar). The y-axis is fixed to −100 to +100%, with dashed red lines at ±40% as pathological thresholds. Brackets with asterisks summarize MD–VM contrasts using two-sided permutation tests (10,000 permutations) with Bonferroni correction across modalities (* p<0.05; ** p<0.01; *** p<0.001; ns = not significant). No legend is shown to maximize plotting area.

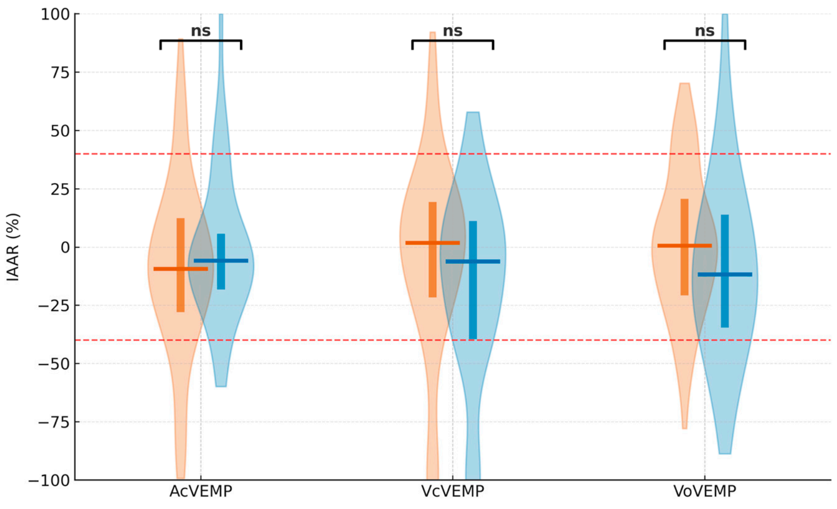

Table S1. IAAR (%) in MD vs VM across VEMP modalities

Variable	Ménière				Migraine				Inference	
	**n**	**mean ± SD**	**median [IQR]**	**% pathol**	**n**	**mean ± SD**	**median [IQR]**	**% pathol**	**p (perm, Bonf.)**	**Sig.**
AcVEMP	55	-8.64 ± 37.21	-9.45 [-26.32, 10.78]	23.6	38	-1.26 ± 31.57	-5.93 [-16.65, 4.12]	15.8	0.9665	ns
VcVEMP	56	-3.81 ± 41.59	1.90 [-20.01, 17.67]	23.2	36	-14.87 ± 39.51	-6.29 [-37.92, 9.58]	25.0	0.6416	ns
VoVEMP	60	0.92 ± 31.07	0.74 [-19.15, 19.08]	20.0	40	-8.69 ± 40.06	-11.79 [-32.93, 12.26]	32.5	0.5540	ns

*Notes. IAAR = interaural amplitude asymmetry ratio. Pathological defined as |IAAR| > 40%. P-values from two-sided permutation tests (10,000 permutations), Bonferroni-adjusted across the three modalities. Significance: *** <0.001; ** <0.01; * <0.05; ns = not significant. MD = Ménière's disease; VM = vestibular migraine; VEMP = vestibular evoked myogenic potential. A: acoustic stimulation; V: vibratory stimulation.*

2. Calibration and Decision-Curve Analysis

Models and validation. We evaluated three prespecified patient-level models excluding PTA to avoid incorporation bias: SDS-only (bilateral SDS), HITCAL-only (caloric–vHIT dissociation, Yes/No), and SDS affected/healthy (exploratory). All models used logistic regression with 5-fold stratified out-of-fold validation; in-fold median imputation and z-standardization were applied to numeric features; HITCAL was one-hot encoded with most-frequent imputation.

Calibration. Out-of-fold probabilities were assessed with calibration curves (10 quantile bins) and summarized by Brier score, calibration slope, and intercept. SDS-only showed the best reliability; SDS affected/healthy was intermediate; HITCAL-only was poorest.

Decision-curve analysis (DCA). Net benefit was computed across thresholds p = 0.05–0.40. SDS-only provided the greatest net benefit across most thresholds, exceeding treat-none and typically treat-all; SDS affected/healthy was intermediate; HITCAL-only showed limited net benefit.

Table S2. Calibration metrics (out-of-fold).

Model	AUC	Brier	Calibration slope	Calibration intercept
SDS-only	0.866	0.167	3.446	0.292
HITCAL-only	0.674	0.193	1.014	0.033
SDS affected/healthy	0.801	0.179	2.450	0.135

Table S3. Decision-curve net benefit by threshold (p = 0.05–0.40).

Threshold	NB: SDS-only	NB: HITCAL-only	NB: SDS affected/healthy	NB: Treat-all	NB: Treat-none
0.05	0.579	0.579	0.579	0.579	0.000
0.10	0.556	0.556	0.556	0.556	0.000
0.15	0.529	0.529	0.529	0.529	0.000
0.20	0.500	0.500	0.500	0.500	0.000
0.25	0.467	0.467	0.467	0.467	0.000
0.30	0.429	0.429	0.429	0.429	0.000
0.35	0.385	0.342	0.385	0.385	0.000
0.40	0.333	0.293	0.333	0.333	0.000

Table S4. HITCAL counts by diagnosis and Fisher's exact test.

Diagnosis	Yes	No	Missing	%Yes (excl. Missing)	Denominator
MD	40	20	0	66.7	60
VM	7	33	0	17.5	40

Fisher's exact test (Yes vs No, excluding Missing): p = 1.56e-06

Figure S2. Calibration (reliability) — SDS-only, HITCAL-only, and SDS affected/healthy.

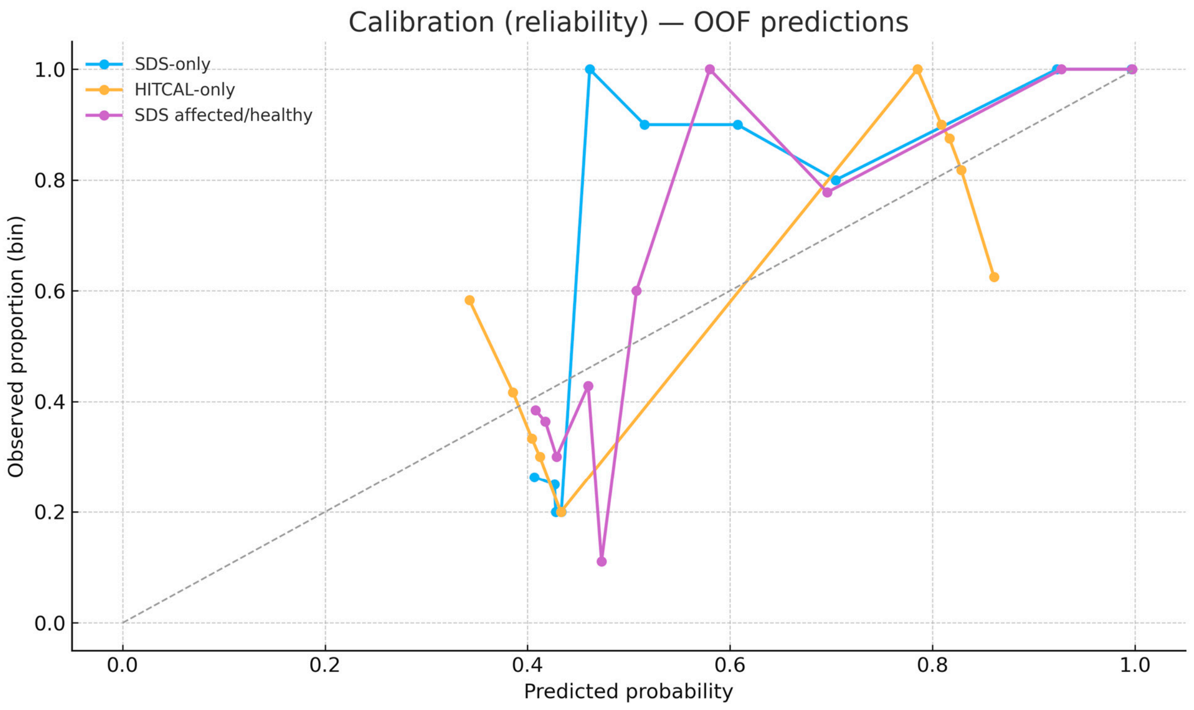

Reliability plots using 10 quantile bins from out-of-fold probabilities. SDS-only aligns best to the identity line; HITCAL-only deviates most. Palette matches the main figures (blue: SDS-only; orange: HITCAL-only; purple: SDS affected/healthy).

Figure S3. Decision-curve analysis — SDS-only, HITCAL-only, and SDS affected/healthy.

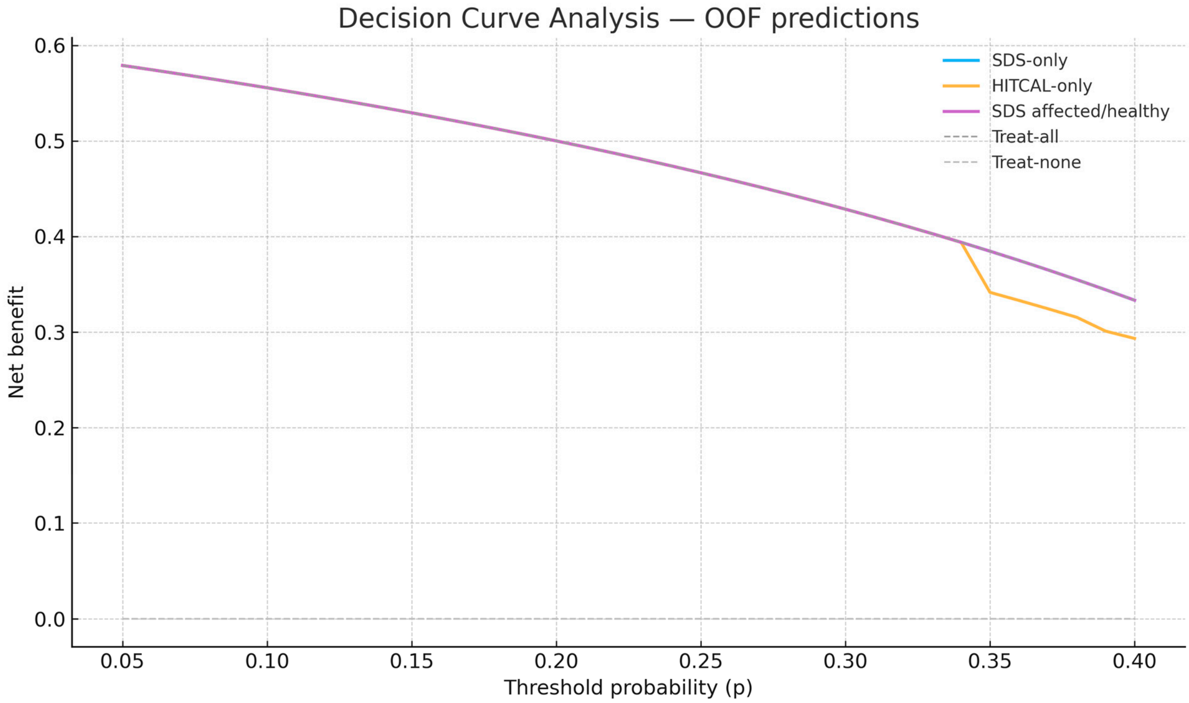

Net benefit across threshold probabilities p = 0.05–0.40 with Treat-all and Treat-none comparators. SDS-only provides the greatest net benefit across most thresholds; HITCAL-only offers limited standalone utility.
